# Phantom Tumours

**Published:** 1909-05-08

**Authors:** 


### MEDICINE.

### PHANTOM TUMOURS.

Skilful palpation of the abdomen requires not only an exact knowledge of the topographical anatomy of the abdominal viscera, of their respective variations in position at different stages of respiration, and of their possible displacements resulting from congenital anomalies or from stretchings of the peritoneal ligaments, but it also requires much personal experience of the varying degrees of rigidity or flaccidity of the muscles of the abdominal wall, of the normal variations in form and consistence that the same organ may exhibit to the palpating hand in different cases, and finally a considerable experience as to which organs can be palpated normally and which can not. Experience alone can enable one to escape the error of sometimes diagnosing as a neoplasm conditions which may be produced in normal or almost normal organs partly by alterations in their proper position and partly by transient, or, at any rate, unimportant changes in their shape or consistence. The weighty question of the sources of error in abdominal palpation is apt to receive too little attention in text-books.

Abdominal tumours may be simulated by the following conditions:—

(1) By abnormal rigidity or tension of the abdominal wall, particularly if only parts of the musculature are involved at a time; (2) by the great omentum; (3) by alterations in the positions of viscera; (4) by undue distension or by abnormality in the contents of the hollow organs; (5) by alterations in the form and condition of the stomach or intestines due to spastic contractions.

It is scarcely necessary to enter into great detail upon the question of mistaking swellings of the abdominal musculature for intra-abdominal tumours; one would like, however, to lay stress upon the lineæ inscriptiones tendineæ of the rectus abdominis muscles, which divide the latter up, as it were, into separate compartments, the portion contained in any one of which may be contracted, when the rest is flaccid, whereby even the most experienced observer may be misled into diagnosing a deep-seated neoplasm or inflammatory mass when there is none. The mistake is particularly liable to occur in nervous patients, especially in women, in whom pregnancy may thus be simulated to a degree of exactness that is surprising. In all such cases much assistance may be gained from getting the patient to cough, whilst the palpating hand notes what happens to the apparent tumour both during and after the effort. Sometimes in the case of a muscular lump it becomes more marked on coughing, whereas an intra-abdominal mass would cease to be palpable when the superficial muscles were strongly contracted. In some cases the only way to be sure is to give a general anæsthetic and then palpate the abdomen when the muscles are thus artificially relaxed.

The kind of condition in which the omentum gives the impression of a tumour to the palpating hand is when parts of it, or the whole of it, have become puckered together, or rolled up in such a way as to simulate either the lower margin of an enlarged liver or else an intra-abdominal malignant tumour. This may result from fibrotic contractions after or during tuberculous peritonitis, or as the result of a former laparotomy. As an instance of the latter may be mentioned the case of a woman of 45 who had had the operation of ventro-fixation of the uterus 12 years before. She had now been suffering for some time past from constant pains in the lower and right side of her abdomen. On deep palpation in this region a distinct tumour could be felt, which was hard, slightly tender to pressure, and but little movable. It seemed to be about the size of the fist. There being some anæmia and loss of weight at the same time, the lump was thought to be malignant, though what the primary site could be was in doubt. An operation having been decided upon and performed, a large portion of the great omentum was found to be rolled up in a most remarkable way into a kind of ball which in turn was firmly united to the parietal peritoneum.

In regard to the third source of errors—congenital and acquired changes in the shape and position of normal organs—the most important examples practically are movable kidney, floating kidney, displaced liver, tight-lacing liver, Riedel's lobe, and wandering spleen. It is not very uncommon to meet with a portion of a "tight-lacing" liver as a freely movable tumour in the abdominal cavity, connected with the main liver merely by a thin bridge of atrophied liver substance or even only fibrous tissue (Riedel's lobe); sometimes this thin connecting band is overlain by bowel, so that one's hand cannot make out any connection between the two parts at all. A wandering spleen may be found in the most variable positions—one day in the right iliac fossa, another in the left, or even deep in the pelvis adjacent to the uterus; and it is clear how difficult the diagnosis may be in some such cases. It is not always possible to decide that the wandering organ is not in its right place, though the effort should always be made to determine this. Helpful points are: the fact that a movable or floating kidney never passes across the middle line, and that it is always better felt bimanually; and that a displaced spleen almost always comes in front of the colon and small intestines, and that it can nearly always be pushed back into its normal site.

As examples of over-distended organs that may give rise to difficulties, the stomach and the bladder may be mentioned. The latter is practically always median; it should disappear on micturition or on the passage of a catheter, but this is not absolutely always so, and it has sometimes given rise to an erroneous diagnosis of pregnancy, whilst, on the other hand, what is really an appendicular abscess in the middle line has been thought to be a distended but otherwise normal bladder. The stomach may be over-distended with either gas or fluid in such a way as to simulate a serious abdominal tumour, of which all signs may have disappeared by next day. The tumours that may be simulated by inspissated fæces in the large intestine are well known to all. The difficulty is a common one. In cases of chronic constipation the nature of a rosary-like series of small, hard lumps beneath the abdominal wall may be obvious; on the other hand, the fæcal mass may be extremely like a carcinoma of the sigmoid flexure, the cæcum, or of one of the flexures. Enteroliths and the like are only special forms of fæcal masses. It is only necessary to add that it by no means follows that a mass is not fæcal when it does not disappear after there has been an abundant evacuation of the bowel.

We may now pass on to the last of the main headings given above—namely, conditions spastic or spasmodic contraction of portions of the alimentary canal simulating tumours.

If one palpates the epigastrium and the region of the pylorus in one and the same individual at different times, it will become clear that, notwithstanding the general teaching that the pylorus is entirely covered by the liver, it can be recognised by palpation far oftener than one might expect—provided, of course, that the muscular development or stoutness of the patient allow of adequate palpation at all. The fact of the pylorus being palpable in this way depends partly upon the thinness of the edge of liver which can readily be pushed up out of the way when deep palpation is being made, but still more upon the motility of the stomach, which produces considerable physiological variations in its topography. When the stomach is empty the pylorus is known to approach the middle line, or even sometimes to lie slightly to the left of the middle line, and as the stomach becomes filled it moves over towards the right. Under certain circumstances, however, the power of the pylorus to "wander" is very much greater than this—in conditions of gastroptosis, for example, or when the organ becomes deformed by gastrectasis. The most extreme degrees of wandering of the pylorus are seen in cases of carcinoma of that end of the stomach; for it may leave the epigastrium entirely, and be found in the hypogastrium or one of the iliac fossæ. The most remarkable example of this fact is recorded by Chiari (*Prager Medizinische Wochenschrift*, 1888), who once met with it in a right-sided inguinal hernia. It is scarcely necessary to insist further upon the importance of this variability in pyloric topography. The forms under which the pylorus presents itself vary with the degree of contraction in the pyloric ring itself, and in the pyloric segment of the stomach. Sometimes it feels like a band, sometimes like a tumour the size of a walnut or a hen's egg, and quite hard; it may extend more or less towards the left according as more or less of the pyloric portion of the stomach assists in the tonic spasm of the pylorus itself. Amongst the various conditions that have been accused of producing phantom tumours from such pyloric spasm may be mentioned mechanical or chemical stimulation by the gastric contents, abnormal quantity or quality of the food, hyperacidity, gastro-succorrhœa, excessive gas formation, ulcers or erosions at or near the pylorus, inflammations around it, and finally influences of a purely functional nervous character. An instance in point is the following: A nervous woman of thirty-seven had for several years suffered from pains in the stomach region, together with vomiting and constipation. The stomach now reached barely to the umbilicus, there was a well-marked succussion splash, and in the epigastrium in the regio pylorica there was to be felt, though not at every examination, a hard tumour about the size of a small apple, movable, tender to pressure, and situated to the right of a readily palpable aorta. The diagnosis was an ulcer at the pylorus with extensive infiltration around it. An operation was performed, and no tumour was to be either seen or felt. After about three minutes, however, the pars pylorica ventriculi passed into so firmly contracted a condition that it constituted a hard tumour-like mass three inches long and nearly two inches thick. There was at the same time a sort of twisting or erection of the contracted portion of the stomach, bringing it closer to the anterior abdominal wall. After about three-quarters of a minute relaxation came on slowly, and the tumour disappeared. Notwithstanding deep chloroform narcosis this phenomenon was repeated three times within a few minutes. There was no macroscopic disease of the stomach at all.

Instances of this kind might be multiplied indefinitely. In children it has sometimes been possible to feel the stomach contracted up into a small epigastric tumour. In the so-called congenital pyloric stenosis a pyloric lump, attributable to spasm, has not infrequently been felt. It may simulate the mass of an intussusception. Whether the spasm is due to hyperacidity or other error of the gastric juice, or whether the errors in the gastric juice are secondary to pyloric spasm, is a question that has never been satisfactorily answered.

How careful one must be in giving an opinion about such spasm-tumours is well exemplified by those cases of simple pyloric ulcer in which, notwithstanding the most careful treatment, hæmorrhage may occur repeatedly, and emaciation may be so great as to suggest that the ulceration is really cancerous. Kampmann (*Strassburger Medizinische Zeitung*, 1907) records the case of a man aged 44, whom he saw in Ewald's wards. This patient had suffered from gastric symptoms on and off for ten years. The diagnosis made was gastro-succorrhœa, with an ulcer at the pylorus. Beneath the edge of the right lobe of the liver in the right para-sternal line, one was able to make out almost constantly a tumour that was tender to pressure, and about the size of a small hen's egg. This, coupled with the facts that the vomit repeatedly contained blood, and that the patient looked decidedly ill and wasted, gave a grave appearance to the case. An operation was performed with the object of possibly being able to remove an early carcinoma. The tumour turned out to be nothing more than the spasmodically contracted pars pylorica of the stomach, with a chronic ulcer on its posterior wall.

The duration of such a spasmodic contraction is very variable. Whereas the mass may last only a few seconds or minutes, and thus be felt obviously relaxing under the palpating fingers, it may, in other cases, persist for hours or even days at a time. Rütimeyer and others have recorded cases in which chronic spasm of the pylorus has remained palpable and apparently unchanged for months. The mere fact that a tumour persists, or appears to persist, from day to day is therefore no absolute proof that the tumour is not a phantom one.

Turning now to a similar state of affairs in the intestines, it is particularly the cæcum, the ascending colon, the transverse colon, and the sigmoid flexure that admit of being palpated through the abdominal parietes; and according to their contents and the degree of spasmodic contraction of their walls they may either not be palpable at all, or else distinctly felt either as soft strands or columns, or as more or less hard tumours. The small intestine is much less often palpable, though entero-spasms may sometimes cause phantom tumours similar to those produced by the colon.

Remarkable tumour-like masses due to spasm can be felt in some cases of chronic colitis, where in addition to mere spasm there is a considerable excess of musculature owing to hypertrophy from former spasms. On either side of the rectus abdominis muscle in the upper part of the abdomen, it is not infrequently possible to feel the corresponding portions of the transverse colon, and it is easy to be led into the belief that there is a well-defined tumour. The apparent clearness of definition of the mass is due to the fact that the ribs cover both the hepatic and the splenic ends of the transverse colon, and the rectus abdominis muscle covers its centre, so that instead of being able to palpate the transverse colon continuously, one is often able only to feel separate portions of it between the ribs and either the left or the right outer border of the upper portion of the rectus abdominis muscle.

Circumscribed spasmodic "knots" in the large intestine, due to persistent local contraction of portions of the bowel, are becoming recognised as a very real cause of difficulty in the diagnosis of acute abdominal conditions, apparently requiring immediate laparotomy, and sometimes the long-maintained spasm of the bowel may produce a definite mass, unilateral and hard, and quite as well-defined as a neoplasm. The lesion is probably an intestinal neurosis. It has led to the performance of many a laparotomy at which no microscopic disease could be found. West records several cases of the kind. Kampmann tells of a tailor who was thirty-five years of age, and highly neurasthenic, and who had been suffering for a year from abdominal pains of the most variable description and from diarrhœa. He thought he had a tumour, but he was kept under observation for some time without any being noticed, until one day there appeared a firm tender lump the size of a hen's egg, apparently in the upper part of the ascending colon, close to the hepatic flexure. It was true that the patient was suffering both from sleeplessness and from the most extreme degree of neurotic phenomena, but the lump seemed real enough. A fæcal mass was excluded by the form and consistence of the lump, and also by the fact that the bowels were being regularly and freely opened. The mass could be made to disappear gradually by persistent circular massage, though it returned again thereafter.

